# Transfusion practices in intensive care units: An Australian and New Zealand point prevalence study

**DOI:** 10.1016/j.ccrj.2023.10.006

**Published:** 2023-12-14

**Authors:** Andrew W.J. Flint, Karina Brady, Erica M. Wood, Le Thi Phuong Thao, Naomi Hammond, Serena Knowles, Conrad Nangla, Michael C. Reade, Zoe K. McQuilten, The George Institute for Global Health, the Australian and New Zealand Intensive Care Society Clinical Trials Group and the Blood Synergy Program

**Affiliations:** a Transfusion Research Unit, Department of Epidemiology and Preventive Medicine, Monash University, Melbourne, Australia; b The Australian and New Zealand Intensive Care Research Centre (ANZIC-RC), School of Public Health and Preventive Medicine, Monash University, Melbourne, Australia; c Royal Australian Navy, Australia; d Intensive Care Unit, Royal Brisbane and Women's Hospital, Herston, Queensland, Australia; e Monash Health, Clayton, Victoria, Australia; f The George Institute for Global Health, Faculty of Medicine, University of New South Wales, Newtown, NSW, Australia; g Malcolm Fisher Department of Intensive Care, Royal North Shore Hospital, Sydney, Australia; h Joint Health Command, Australian Defence Force, Canberra, Australia; i Faculty of Medicine, University of Queensland, Brisbane, Australia

**Keywords:** 4 Anaesthesia and Intensive care, 4.24 Intensive care, 21 Haematology

## Abstract

**Objective:**

To describe current transfusion practices in intensive care units (ICUs) in Australia and New Zealand, compare them against national guidelines, and describe how viscoelastic haemostatic assays (VHAs) are used in guiding transfusion decisions.

**Design, setting and participants:**

Prospective, multicentre, binational point-prevalence study. All adult patients admitted to participating ICUs on a single day in 2021.

**Main outcome measures:**

Transfusion types, amounts, clinical reasons, and triggers; use of anti-platelet medications, anti-coagulation, and VHA.

**Results:**

Of 712 adult patients in 51 ICUs, 71 (10%) patients received a transfusion during the 24hr period of observation. Compared to patients not transfused, these patients had higher Acute Physiology and Chronic Health Evaluation II scores (19 versus 17, *p* = 0.02), a greater proportion were mechanically ventilated (49.3% versus 37.3%, *p* < 0.05), and more had systemic inflammatory response syndrome (70.4% versus 51.3%, *p* < 0.01). Overall, 63 (8.8%) patients received red blood cell (RBC) transfusions, 10 (1.4%) patients received platelet transfusions, 6 (0.8%) patients received fresh frozen plasma (FFP), and 5 (0.7%) patients received cryoprecipitate. VHA was available in 42 (82.4%) sites but only used in 6.6% of transfusion episodes when available. Alignment with guidelines was found for 98.6% of RBC transfusions, but only 61.6% for platelet, 28.6% for FFP, and 20% for cryoprecipitate transfusions.

**Conclusions:**

Non-RBC transfusion decisions are often not aligned with guidelines and VHA is commonly available but rarely used to guide transfusions. Better evidence to guide transfusions in ICUs is needed.

## 1 Introduction

Anaemia, thrombocytopenia, and coagulopathy occur commonly in intensive care unit (ICU) patients and are associated with poor outcomes.[1], [2], [3], [4], [5] Transfusions are often administered in these patients to improve oxygen delivery or correct coagulopathy and, when used appropriately, are potentially lifesaving. All transfusions do, however, carry risks and costs.[6], [7], [8]

**Fig. 1:**
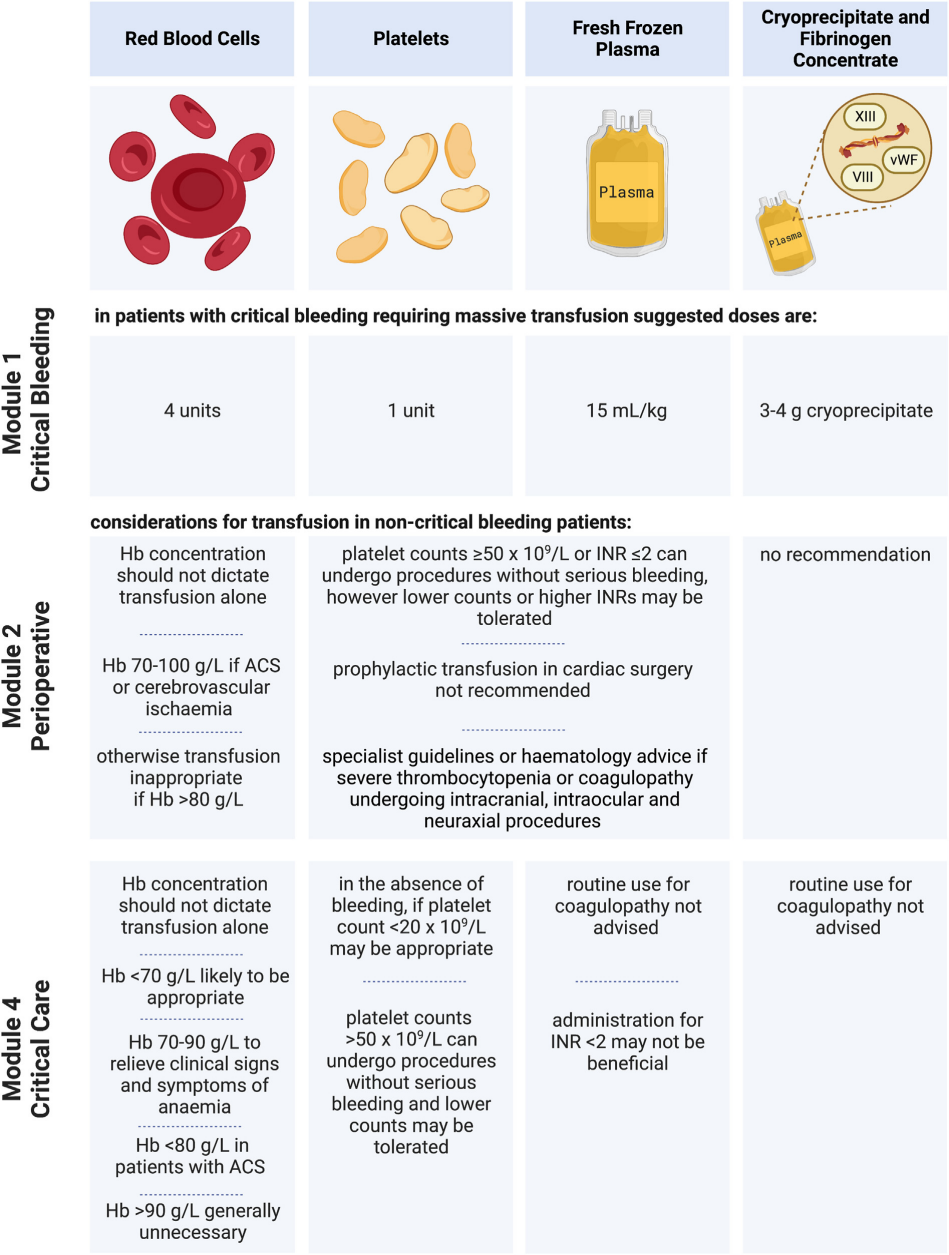
Summaries of the National Blood Authority's Patient Blood Management guidelines.^9^^,^^10^^,^^20^ Transfusion practice was not compared against the Paediatric/Neonatal or Obstetric/Maternity guidelines, because all patients were adults, and no patients were reported to be pregnant. Created with BioRender.com.

The decision to transfuse can be difficult and may be based on clinical features, laboratory values, and/or viscoelastic haemostatic assay (VHA) results. Australia's National Blood Authority Patient Blood Management (PBM) guidelines (the PBM guidelines) were developed to guide transfusion decisions and include modules on critical care, critical bleeding/massive transfusion, and perioperative transfusions.^9^^,^^10^ The summaries of the PBM guidelines are given in Fig. 1. However, for many aspects of transfusion practice in ICU, the PBM guidelines could not make evidence-based recommendations due to lack of data.

The strongest evidence is for red blood cell (RBC) transfusions, with randomised controlled trials (RCTs) demonstrating equivalence of restrictive compared to liberal transfusion strategies.[11], [12], [13] However, there are instances where a more liberal transfusion strategy might be preferred, such as patients with acute coronary syndrome (ACS), cardiovascular disease, and elderly patients.[14], [15], [16] The optimal transfusion strategy in ICU is less well established for platelet, fresh frozen plasma (FFP), cryoprecipitate, and fibrinogen transfusions.

Prior to the publication of the PBM guidelines, the Australian and New Zealand (ANZ) Blood Observational Study performed across 47 ICUs over a five-week period in 2008 found that while most RBC transfusions were given according to guidelines, approximately 53% of platelet transfusions, 29% FFP, and 88% of cryoprecipitate transfusions were not.^17^

Since the introduction of the PBM guidelines, there has been no large, multi-centre evaluation of transfusion practices in ANZ ICUs and there have been clinical changes which may have altered transfusion practice. Specifically, the use of VHA results to guide transfusion decisions is increasingly reported; however, the evidence base to support its use remains uncertain.^18^ How common VHAs are and how it is used in ICUs is not known.

The aims of this study were to describe current transfusion practices in ICUs across ANZ, compare them against previous transfusion practice and the PBM guidelines, and to determine whether and how VHA are being used in transfusion decision-making.

## 2 Methods

This was a prospective, multicentre, point-prevalence study of ICU patients. Data were collected through the Point Prevalence Program of the Australian and New Zealand Intensive Care Society Clinical Trials Group (ANZICS-CTG) and the George Institute for Global Health. Participating ICUs collected 24 h of data for all admitted patients on one of two study days in June 2021, with 28-day follow-up.

We collected data using REDCap (Vanderbilt University, Nashville, United States of America (USA)) hosted at The George Institute for Global Health.^19^ Data collected included patient demographics, transfusion-related data and outcome data. Patient demographics included age, gender, Acute Physiology and Chronic Health Evaluation (APACHE II) score, admission diagnosis, ICU and hospital admission dates, and ICU admission source. Transfusion-related data included types and number of units transfused, clinical values that triggered transfusion, main clinical indication for transfusion, and relevant pre- and post-transfusion laboratory values. We did not collect data on transfusion reactions. The definition of major bleeding was taken from the PBM Guidelines to be life-threatening bleeding likely to result in the need for massive transfusion, whereas minor bleeding was non-life-threatening bleeding.^10^ We compared transfusion practice against recommendations made in the PBM Guidelines’ Module 1 Critical Bleeding/Massive Transfusion, Module 2 Perioperative and Module 4 Critical Care, according to whichever guideline was most relevant for the clinical indication.^9^^,^^10^^,^^20^ Additionally, we collected data on anti-platelet and anti-coagulation medications administered on the study day and prior to ICU admission. VHA data were collected at the unit level and included the availability of VHAs, type, and its location.

A transfusion of blood products was considered to constitute a RBC, platelet, FFP, or cryoprecipitate transfusion—other blood products were considered separately. A transfusion episode was defined as one or more transfusions requested at the same time, regardless of the time of administration. A massive transfusion was considered to be either ≥ 10 units of RBCs in 24 h, ≥ 6 units in 6 h, or ≥ 5 units in 4 h, based on commonly used definitions of massive transfusions.^21^

Descriptive statistics were reported according to data distribution as either mean (standard deviation [SD]) or median (interquartile range [IQR]). We compared transfused patients to non-transfused patients using Student's t-test for continuous variables and chi-square for categorical variables. We investigated variability across sites in several ways by grouping sites according to country, ICU level, metropolitan versus rural, and public versus private ICU; adjusting each model for APACHE II score. A two-sided significance level of *p* < 0.05 was considered to indicate statistical significance. STATA 15 (College Station, Texas, USA) was used for all statistical analysis.^22^

Human research ethics committee or institutional approval for a waiver of individual patient consent was obtained for all participating sites.

## 3 Results

Fifty-one ICUs participated in one of two study dates in June 2021. A breakdown of site characteristics is given in Table 1—ICU level is reported according to the College of Intensive Care Medicine minimum standards for ICUs, where Level III is a tertiary referral unit.^23^

**Table 1:** Site characteristics (number of sites, %).

Total sites	51
Country	
*Australia*	40 (78.4)
*NZ*	11 (21.6)
ICU level	
*Level III*	34 (66.7)
*Level II*	16 (31.4)
*Level I*	1 (2.0)
Metropolitan or rural/remote	
*Metropolitan*	46 (90.2)
*Rural or remote*	5 (9.8)
Public or private	
*Public*	45 (88.2)
*Private*	5 (9.8)
*Combined*	1 (2.0)

Seven-hundred and twelve adult patients were included in the study. The mean age was 60 (SD 17) years, and the majority (62.9%) were male (Table 2).

**Table 2:** Characteristics of transfused and non-transfused patients^a^.

	All patients (n = 712)	Transfused patients (n = 71)	Patients not transfused (n = 641)	P value
Age, years	60.4 (17.0)	60.3 (14.8)	60.4 (17.2)	0.95
Gender, male	448 (62.9)	46 (64.8)	402 (62.7)	0.73
BMI, kg/m^2^	29.8 (9.8)	28.1 (10.2)	30.0 (9.7)	0.15
APACHE II score	17 (8)	19 (7)	16.7 (7.5)	0.02
ICU admission source				0.06
*Elective surgery*	136 (19.1)	17 (23.9)	119 (18.6)	
*Emergency surgery*	106 (14.9)	12 (16.9)	94 (14.7)	
*Emergency department*	222 (31.2)	15 (21.1)	207 (32.3)	
*Hospital ward*	163 (22.9)	19 (26.8)	144 (22.5)	
*Transfer from another ICU*	38 (5.3)	7 (9.9)	31 (4.8)	
*Transfer from another hospital (not ICU)*	47 (6.6)	1 (1.4)	46 (7.2)	
Primary ICU non-operative diagnosis				0.10
*Cardiovascular*	84 (18.2)	9 (22.5)	75 (17.8)	
*Respiratory*	97 (21.0)	6 (15.0)	91 (21.6)	
*Gastrointestinal*	35 (7.6)	6 (15.0)	29 (6.9)	
*Neurological*	68 (14.8)	2 (5.0)	66 (15.7)	
*Sepsis*	74 (16.1)	5 (12.5)	69 (16.4)	
*Trauma and burns*	45 (9.8)	3 (7.5)	42 (10.0)	
*Metabolic*	26 (5.6)	5 (12.5)	21 (5.0)	
*Haematological*	8 (1.7)	2 (5.0)	6 (1.4)	
*Renal/genitourinary*	11 (2.4)	0 (0.0)	11 (2.6)	
*Gynaecological*	3 (0.7)	2 (5.0)	66 (15.7)	
*Musculoskeletal/skin*	6 (1.3)	1 (2.5)	5 (1.2)	
*Other medical*	4 (0.9)	1 (2.5)	3 (0.7)	
Post-operative admission	251 (35.3)	31 (43.7)	220 (34.3)	0.12
Primary ICU operative diagnosis				0.33
*Cardiovascular*	55 (21.9)	8 (25.8)	47 (21.4)	
*Respiratory and ENT*	19 (7.6)	2 (6.5)	17 (7.7)	
*Gastrointestinal*	55 (21.9)	8 (25.8)	47 (21.4)	
*Neurological*	31 (12.4)	0 (0.0)	31 (14.1)	
*Trauma and burns*	25 (10.0)	3 (9.7)	22 (10.0)	
*Renal/genitourinary*	9 (3.6)	1 (3.2)	8 (3.6)	
*Gynaecological*	6 (2.4)	0 (0.0)	6 (2.7)	
*Musculoskeletal/skin*	14 (5.6)	1 (3.2)	13 (5.9)	
Invasive mechanical ventilation	274 (38.5)	35 (49.3)	239 (37.3)	<0.05
Respiratory support	210 (29.5)	20 (28.2)	190 (29.6)	
ARDS	10 (1.4)	2 (2.8)	8 (1.3)	0.29
Injury by blunt or penetrating trauma	78 (11.0)	7 (9.9)	71 (11.1)	0.76
Defined focus of infection	247 (34.7)	29 (40.9)	218 (34.0)	0.25
SIRS	379 (53.2)	50 (70.4)	329 (51.3)	<0.01
Vasopressors to maintain MAP	235 (33.0)	38 (53.5)	197 (30.7)	<0.01
Chronic health conditions				
*Cirrhosis and portal hypertension or previous hepatic failure*	23 (3.2)	5 (7.0)	18 (2.8)	0.06
*NYHA IV*	22 (3.1)	4 (5.6)	18 (2.8)	0.19
*Chronic respiratory disease*	55 (7.7)	4 (5.6)	51 (8.0)	0.49
*Immunocompromised*	91 (12.8)	11 (15.5)	80 (12.5)	0.47
Medications				
Anti-platelet medication prior to ICU admission	189 (27.5)	16 (23.9)	173 (27.9)	0.49
Anti-platelet medication during ICU admission	198 (27.9)	22 (31.0)	176 (27.5)	0.53
Anti-coagulation medication prior to ICU admission	172 (25.3)	20 (29.9)	152 (24.8)	0.36
Anti-coagulation medication during ICU admission	482 (67.9)	44 (62.0)	438 (68.5)	0.26

APACHE, acute physiology and chronic health evaluation; ARDS, acute respiratory distress syndrome; BMI, body mass index; ENT, ear, nose and throat; ICU, intensive care unit; MAP, mean arterial pressure; NYHA, New York Heart Association; SIRS, systemic inflammatory response syndrome.

a Results are reported in mean (SD) for continuous variables and n (%) for categorical variables.

Seventy-one (10.0%) patients received a transfusion consisting of 87 transfusion episodes. Compared to ICU patients not transfused, patients who received a transfusion had higher admission APACHE II scores, and on the study day, a greater proportion were mechanically ventilated, administered vasopressors, and met the criteria for systemic inflammatory response syndrome. Only three (0.5%) patients had confirmed or suspected SARS-CoV-2 Infection.

### 3.1 RBC transfusions

There were 73 RBC transfusion episodes (83.9% of transfusion episodes), with a median of one (IQR 0) unit per transfusion episode, and a maximum of 24 RBC units. Sixty-three out of 712 patients (8.8%) received RBC transfusions and 12 patients (1.7%) received multiple RBC transfusions—the median number of units received by patients receiving RBCs was 1 (IQR 1), with a maximum of 25 RBC units.

The reasons for RBC transfusion were known for 65 (89.0%) of the 73 RBC transfusion episodes (Table 3). Sixty-eight (93.2%) RBC transfusion episodes had an Hb transfusion trigger documented. The Hb transfusion triggers for RBC transfusion episodes was <70 g/L for 15 (20.6%) episodes, 70–79 g/L for 43 (58.9%) episodes, and 80–89 g/L for 10 (13.7%) episodes. Of the remaining five RBC transfusions without a documented trigger, one had a Hb > 90 g/L and this patient was not recorded as having a cardiovascular admission diagnosis or condition, and was only reported to have minor bleeding, and this was, therefore, likely to be inconsistent with the PBM guidelines; one was for major bleeding and, therefore, consistent with the guidelines; the remaining three had Hb levels ≤ 90 g/L and were assumed to be consistent with the PBM guidelines based on clinically significant anaemia and/or ACS. In total, 71 out of 72 (98.6%) RBC transfusion episodes were considered likely to be consistent with the PBM guidelines.

**Table 3:** Number of clinical reasons (%) for RBC, platelet, FFP and cryoprecipitate transfusions.

Clinical reason	RBC	Platelet	FFP	Cryoprecipitate
Major bleeding	5 (6.9)	2 (15.4)	2 (28.6)	2 (40.0)
Minor bleeding	25 (34.3)	2 (15.4)	–	1 (20.0)
Anaemia/improve oxygen delivery	22 (30.1)	1 (7.7)	1 (14.3)	
Coagulopathy	6 (8.2)	2 (15.4)	3 (42.9)	1 (20.0)
Prophylaxis against spontaneous bleeding^a^	1 (1.4)	1 (7.7)	–	1 (20.0)
Prophylaxis for an invasive procedure	4 (5.5)	2 (15.4)	–	–
Other^b^	2 (2.7)	–	1 (14.3)	–
Unknown	8 (11.0)	3 (23.1)	–	–
Total	73	13	7	5

FFP, fresh frozen plasma; RBC, red blood cell.

a Invasive procedures included (but were not limited to) insertion and removal of invasive lines and devices.

b Other included, RBC: Low haematocrit on ECMO, sepsis, FFP: plasmapheresis.

The mean Hb prior to RBC transfusion was 75 (SD 7) g/L. The mean Hb prior to RBC transfusion was lower in those who received multiple RBC transfusions (68; SD 5 g/L). Following transfusion of one RBC unit, the mean increase in Hb was 10 (SD 10) g/L.

There were no significant differences between the proportions of patients receiving anti-platelet or anticoagulation medication either prior to or during their ICU admission, for the RBC and non-RBC groups.

### 3.2 Platelet transfusions

Thirteen transfusion episodes involved platelets (14.9% of 87 transfusion episodes; median one unit, IQR 0). Ten out of 712 patients (1.4%) received platelet transfusions with a median of one (IQR 1) unit, and three patients received multiple platelet transfusions.

A clinical indication for platelet transfusion was known for 10 (76.9%) of the 13 platelet transfusion episodes (Table 3). Thirteen (100%) platelet transfusion episodes had a documented platelet count trigger. Eight out of 13 transfusion episodes (61.5%) were consistent with guidelines with four transfusion triggers (30.8%) <20 × 10^9^/L, two transfusion triggers (15.4%) 20–50 × 10^9^/L and undergoing an invasive procedure, and two transfusion triggers (15.4%) ≥ 20 × 10^9^/L but indicated for major bleeding. For the remaining five (38.4%) platelet transfusion episodes, the platelet count trigger was ≥ 20 × 10^9^/L and not documented to be undergoing a procedure or have major bleeding as the indication, which was not consistent with the PBM guidelines.

The mean pre-transfusion platelet count overall was 59 (SD 84) × 10^9^/L. This was lower in those undergoing procedures than those not undergoing procedures (31 versus 64 × 10^9^/L) and higher in bleeding patients than non-bleeding patients (88 versus 46 × 10^9^/L). The mean increase in platelet count following one unit was 18 (SD 19) × 10^9^/L.

No patients who received platelet transfusions were reported to have received anti-platelet medications prior to or during their ICU admission. While two (20%) out of 10 patients who received platelet transfusions were on anti-coagulation medication prior to ICU admission, no patients who received platelets remained on anti-coagulation during their ICU admission.

### 3.3 Fresh frozen plasma transfusions

Seven of the 87 transfusion episodes (8.0%) involved FFP, with a median of two (IQR 0) units. Six out of 712 patients (0.8%) received FFP with a median of two (IQR 0) units.

The clinical reason for transfusion was known for all FFP transfusion episodes (Table 3). Three out of seven FFP transfusion episodes (42.9%) were consistent with the PBM guidelines: two for major bleeding and we considered the FFP transfusion for plasmapheresis to be appropriate even though not explicitly mentioned in the PBM guidelines. Four out of seven FFP transfusions episodes (57.1%) were not transfused according to the PBM guidelines, as they were transfused with an international normalised ratio (INR) < 2 and/or without major bleeding or procedural related reasons.

A trigger for FFP transfusion was given for all FFP transfusion episodes. An elevated INR was the trigger for four FFP transfusions (57.1%), with a mean INR trigger of 1.5 (SD 0.1). An elevated activated partial thromboplastin time (APTT) of 47 s and VHAs were also used as triggers for FFP transfusion.

The mean pre-FFP transfusion INR and APTT were 1.4 (SD 0.2) and 41 (SD 6.2) seconds, respectively. The mean decreases in INR and APTT following FFP transfusion were 0.14 (SD 0.2) and 8.2 (SD 8.1) seconds, respectively.

There were no significant differences in the proportions of patients receiving FFP transfusions who were on anti-platelet or anti-coagulation medications prior to or during ICU admission.

### 3.4 Cryoprecipitate and fibrinogen concentrate

Five of the 87 transfusion episodes (5.7%) involved cryoprecipitate, with a median of six (IQR 6) units per transfusion episode. Five patients out of 712 (0.7%) received cryoprecipitate, and they received a median of six (IQR 8) units. It is unknown whether these were whole blood-derived or apheresis cryoprecipitate units. No transfusions episodes involved fibrinogen concentrate.

The clinical indication for cryoprecipitate transfusion was known for all transfusion episodes, with major bleeding being the most common indication (Table 3). Fibrinogen level was documented as the trigger for cryoprecipitate transfusion in all transfusion episodes, with a median of 1.8 g/L (IQR 1.1). It is unknown if any of these patients were pregnant with higher expected fibrinogen levels. The mean increase in fibrinogen level post-cryoprecipitate transfusion was 0.8 (SD 0.3). VHA results were used in conjunction with fibrinogen level as the trigger for one patient with minor bleeding.

In contrast to the PBM guidelines, three of the five cryoprecipitate transfusion episodes (60%) were transfused without an indication of major bleeding, and all of these patients had fibrinogen levels within normal ranges pre-transfusion; the remaining two out of five (40%) were for major bleeding and consistent with the PBM guidelines.

Comparing patients who received cryoprecipitate transfusions to those who did not, there were no significant differences in the proportions of patients who received anti-platelet or anti-coagulation mediation prior to or during ICU admission.

### 3.5 Other blood products

Three out of 712 patients (0.4%) received immunoglobulin. No patients received recombinant factor VIIa.

### 3.6 Massive transfusion

One out of 712 patients (0.1%) patient received a massive transfusion, which included 25 units of RBCs within 4 h. They also received one unit of platelets within the same time frame.

### 3.7 Anti-platelet and anti-coagulation medications

The proportions of patients who received anti-platelet and anti-coagulation medications are shown in Tables A and B of the supplemental material, respectively. There were no significant differences in proportions of patients who received a transfusion for patients on anti-platelet or anti-coagulation medications.

### 3.8 Viscoelastic haemostatic assays

VHAs were available at 42 out of 51 sites (82.4%) and, of these, available within the ICU at 27 (64.3%) sites. At one site, the VHA was unavailable on the study day. The system used was known at 35 sites and was rotational thromboelastometry (ROTEM) at 21 (60%) and thromboelastography (TEG) at 14 (40%) of these sites. At sites where a VHA was available, VHA results were used in five of their 76 transfusion episodes (6.6%) and two of their 21 non-RBC transfusion episodes (9.5%).

### 3.9 Site variability

Table 4 shows transfusion types across different site categories. After adjusting for APACHE II score, whether a patient received any type of transfusion was not significantly associated with Australia versus New Zealand (odds ratio (OR) 2.1, 95% confidence interval (CI) 0.86 to 4.86), tertiary hospital versus non-tertiary (OR 1.4, 95% CI 0.6 to 3.2), rural versus metropolitan (OR 0.8, 95% CI 0.2 to 3.5), or public versus private (OR 1.1, 95% CI -0.3 to 2.6).

**Table 4:** Number (%) of patients transfused for each site category.

	Total number of patients	Any transfusion	RBC	Platelet	FFP	Cryoprecipitate
Country						
*Australia*	598	65 (10.9)	58 (9.7)	10 (1.7)	5 (0.8)	5 (0.8)
*New Zealand*	114	6 (5.3)	5 (4.4)	0	1 (0.9)	0
Unit level						
*Level I*	6	0	0	0	0	0
*Level II*	95	7 (7.4)	5 (5.3)	0	2 (2.1)	2 (2.1)
*Level III*	611	64 (10.5)	58 (9.5)	10 (1.6)	4 (0.65)	3 (0.5)
Rurality						
*Rural*	26	2 (7.7)	0	0	1 (3.9)	1 (3.9)
*Metropolitan*	686	69 (10.1)	63 (9.2)	10 (1.5)	5 (0.7)	4 (0.6)
Public/private						
*Public*	651	69 (10.6)	61 (9.4)	10 (1.5)	6 (0.9)	5 (0.8)
*Private*	47	1 (2.1)	1 (2.1)	0	0	0
*Combined*	14	1 (7.1)	1 (7.1)	0	0	0

FFP, fresh frozen plasma; RBC, red blood cell.

## 4 Discussion

This is the first study to evaluate ICU transfusion practices in ANZ since the Blood Observational Study of 2010 and publication of the PBM guidelines.^9^^,^^10^^,^^17^ We found that only 10% of ICU patients received a transfusion of blood components and these patients were more severely ill. A laboratory trigger for transfusion was often reported, and while VHAs were available at most sites and frequently within the ICU itself, it was only used to guide a small number of transfusion episodes, more commonly for non-RBC transfusions. Overall, a majority of transfusions and nearly all RBC transfusions were aligned with the PBM guidelines; however, transfusion of non-RBC transfusions often was not.

A comparison to historical transfusion practices in ANZ is shown in Table 5 using results from the Blood Observational Study.^17^ Compared to the Blood Observational Study, we found lower rates of transfusions and the proportions of patients who received platelets, FFP, and cryoprecipitate to be lower. We also found lower haemoglobin concentration and platelet count triggers for RBC and platelet transfusions, respectively. These results may indicate a change in practice towards more restrictive transfusion strategies, which was also reported in the Blood Observational Study and might represent an overall long-term trend towards restrictive transfusion strategies. In contrast to this, however, more liberal triggers were found for FFP and cryoprecipitate transfusions, but these were not for procedural related reasons. Similarly to the Blood Observational Study, we found a high level of agreement with guidelines for RBC transfusions, but not for non-RBC transfusions—this may reflect the lack of evidence to guide recommendations for non-RBC transfusion, whereas the evidence for areas of RBC transfusion is more established.[11], [12], [13] For instance, at the time the study was conducted, no large RCTs of platelet or cryoprecipitate transfusions in ICU patients had been performed, and the only RCT of FFP in critically ill patients was abandoned due to slow recruitment.^24^

**Table 5:** Comparison of key results to the Blood Observational Study.

	2010 Blood Observational Study	2021 Point Prevalence Study
Number of patients	874	712
Proportion of patients receiving blood transfusions	17.0%	10.0%
Percentage of transfused patients receiving:		
- RBC	86.6%	88.7%
- Platelets	38.9%	14.1%
- FFP	26.4%	8.5%
- Cryoprecipitate	8.9%	7.0%
Anti-platelet agents transfused patients in ICU		
- Aspirin	36.8%	29.6%
- Clopidogrel	10.4%	5.6%
Mean pre-RBC transfusion Hb levels	77.6 g/L	73.7 g/L
Proportion of RBC transfusions with Hb < 70 g/L	19.0%	32.9%
Mean pre-platelet transfusion platelet count	67.8 x 10^9^/L	58.7 x 10^9^/L
Proportion of platelet transfusion platelet counts >50 x 10^9^/L	52.8%	23.1%
Mean pre-FFP transfusion INR	1.8	1.4
Mean pre-cryoprecipitate fibrinogen level	1.4 g/L	2.3 g/L
Consistency with national guidelines:		
- RBC	98%	99%
- Platelets	47%	62%
- FFP	71%	43%
- Cryoprecipitate	12%	40%

FFP, fresh frozen plasma; Hb, Haemoglobin; ICU, intensive care unit; INR, international normalised ratio; RBC, red blood cell.

This study has several limitations. We only captured 24 h of data in ICU and, therefore, may have missed important transfusion data occurring outside the study period and ICU admission. Additionally, by performing a point prevalence study, we were not able to accurately capture less common interventions and this might explain: why we did not record any instances of fibrinogen concentrate and recombinant factor VIIa, the low numbers of non-RBC transfusions, and why percentages of platelet transfusions were lower than other studies of platelet transfusions in ICU where platelet transfusion rates were approximately 10%.^4^^,^^6^ Relatively infrequent non-RBC transfusions made it difficult to accurately determine their true consistency with the PBM guidelines, and these overall results were more likely to be affected by site variation or documentation issues, including inaccurate data because the data was not entered by the prescribing clinician. The short time period also made it difficult to measure accurately massive transfusions, with definitions of massive transfusions usually based on the number of RBC units per unit time and commonly up to 24 h. Another limitation is that comparing transfusion triggers alone with PBM guidelines oversimplifies the decision-making process and transfusions, we found to be inconsistent with guidelines, may well have been appropriate in their clinical setting because we did not collect all clinically important data, such as the presence of ACS, symptomatic anaemia, or administration of extracorporeal membrane oxygenation. Conversely, we assumed clinicians administered appropriate transfusions in some circumstances where we did not have all information, such as RBC transfusions in the Hb 70–90 g/L range, assuming the patients must have had signs or symptoms of anaemia or ACS. In addition, the lack of evidence in particular for non-RBC transfusions in ICU was a limitation to our capacity to evaluate current standards of practice. A limitation of our VHA analysis was we did not capture how often VHAs were used when considering non-RBC transfusions but resulted in the decision not to transfuse. Finally, a shortcoming of the case report form was that it did not ask specifically for details about FFP transfusions, and it relied upon researchers to report FFP under *other transfusions*—this might account partly for the lower number of FFP transfusions recorded.

The strengths of this study are that it was a prospective, multicentre, bi-national study of transfusion practices in the ICU involving a heterogeneous adult population of medical and surgical patients. There were only three patients with known or suspected SARS-CoV-2 infection and results therefore represent pre-pandemic transfusion practices, which is important as the impact of SARS-CoV-2 on transfusion practices are unknown.

Future large observational studies over a longer period are required to confirm our findings. Knowledge of the benefit of platelet, FFP, and cryoprecipitate transfusions for patients in ICU is lacking, requiring future RCTs in critically ill patients to inform transfusion decisions and future guidelines. Furthermore, seeking evidence for the role and benefit of VHAs in the ICU is an important area of future research.

## 5 Conclusion

In ICUs of ANZ, RBC transfusions are mostly consistent with national guidelines but non-RBC transfusions commonly are not. VHAs are often available but are rarely used as the trigger for transfusions. Given the costs and the potential harms of inappropriate transfusions in ICU patients, more evidence about which patients benefit from transfusions is required.

## CRediT authorship contribution statement

AF, KB, EW, MR and ZM were involved with the application for funding, and the concept and design of the study. NH, SK, CN were responsible for running the point prevalence program, data collection and data cleaning. AF and LT produced the results and statistical analysis. AF and KB developed the figure. All authors were involved in the interpretation of the results, and drafting and the final review of the manuscript.

## Conflict of interest

The authors declare the following financial interests/personal relationships which may be considered as potential competing interests: Andrew Flint reports financial support was provided by Australian and New Zealand Society of Blood Transfusion. If there are other authors, they declare that they have no known competing financial interests or personal relationships that could have appeared to influence the work reported in this paper.
