# Supplementary material for: Transfusion practices in intensive care units: An Australian and New Zealand point prevalence study
Source: Crit Care Resusc. 2023 Dec 14;25(4):193–200. doi: 10.1016/j.ccrj.2023.10.006 (PMC10790088; doi:10.1016/j.ccrj.2023.10.006)
Supplement: Multimedia component 1 [file mmc1.docx]

**Supplementary Material**

Table A: Number of patients (% of N = 712) who received anti-platelet medications prior to and during ICU admission ^a^

|  | Anti-platelet medications | |
| --- | --- | --- |
|  | Prior to ICU admission 189 (26.5) | During ICU admission 198 (27.8) |
| *Aspirin* | 169 (23.7) | 186 (26.1) |
| *Clopidogrel* | 34 (4.8) | 26 (3.7) |
| *Ticagrelor* | 14 (2.0) | 10 (1.4) |
| *Prasugrel* | 0 | 1 (0.1) |
| *Tirofiban* | 0 | 0 |
| *Other NSAID* | 2 (0.3) | 1 (0.1) |
| *Other agent* | 0 | 0 |
| Dual anti-platelet agents | 30 (4.2) | 26 (3.6) |
| No anti-platelet agent | 499 (70.1) | 513 (72.1) |
| Unknown | 24 (3.4) | 1 (0.1) |

^a^ Percentages of total patients (N = 712). One patient may receive multiple agents.

NSAID, non-steroidal anti-inflammatory drug.

Table B: Number of patients (% of N = 712) who received anti-coagulation medications prior to and during ICU admission ^a^

|  | Anti-coagulation medications | |
| --- | --- | --- |
|  | Prior to ICU admission 172 (24.2) | During ICU admission 482 (67.7) |
| Prophylactic anti-coagulation | 65 (9.1) |  |
| *Heparin* | 22 (3.1) | 210 (29.5) |
| *Enoxaparin* | 43 (6.0) | 177 (24.9) |
| Therapeutic anticoagulation | 123 (17.3) |  |
| *Heparin* | 32 (4.5) | 39 (5.5) |
| *Enoxaparin* | 17 (2.4) | 26 (3.7) |
| *Warfarin* | 20 (2.8) | 15 (2.1) |
| *Rivaroxaban* | 20 (2.8) | 10 (1.4) |
| *Apixaban* | 24 (3.4) | 9 (1.3) |
| *Dabigatran* | 5 (0.7) | 1 (0.1) |
| *Other agent* | 5 (0.8) | 8 (1.1) |
| No anti-coagulation | 509 (72.5) | 228 (32.0) |
| Unknown | 31 (4.4) | 2 (0.3) |

^a^ Percentages of total patients (N = 712). One patient may receive multiple agents.
